# The Role of APOE and TREM2 in Alzheimer′s Disease—Current Understanding and Perspectives

**DOI:** 10.3390/ijms20010081

**Published:** 2018-12-26

**Authors:** Cody M. Wolfe, Nicholas F. Fitz, Kyong Nyon Nam, Iliya Lefterov, Radosveta Koldamova

**Affiliations:** Department of Environmental & Occupational Health, University of Pittsburgh, Pittsburgh, PA 15261, USA; cody.wolfe@pitt.edu (C.M.W.); nffitz@pitt.edu (N.F.F.); Kyn5@pitt.edu (K.N.N.)

**Keywords:** Alzheimer’s disease (AD), Apolipoprotein E (APOE), triggering receptor expressed on myeloid cells 2 (TREM2), *tyrobp*, microglia, inflammation, amyloid beta, amyloidogenesis

## Abstract

Alzheimer’s disease (AD) is the leading cause of dementia worldwide. The extracellular deposits of Amyloid beta (Aβ) in the brain—called amyloid plaques, and neurofibrillary tangles—intracellular tau aggregates, are morphological hallmarks of the disease. The risk for AD is a complicated interplay between aging, genetic risk factors, and environmental influences. One of the Apolipoprotein E (*APOE*) alleles—*APOEε4*, is the major genetic risk factor for late-onset AD (LOAD). APOE is the primary cholesterol carrier in the brain, and plays an essential role in lipid trafficking, cholesterol homeostasis, and synaptic stability. Recent genome-wide association studies (GWAS) have identified other candidate LOAD risk loci, as well. One of those is the triggering receptor expressed on myeloid cells 2 (*TREM2*), which, in the brain, is expressed primarily by microglia. While the function of TREM2 is not fully understood, it promotes microglia survival, proliferation, and phagocytosis, making it important for cell viability and normal immune functions in the brain. Emerging evidence from protein binding assays suggests that APOE binds to TREM2 and APOE-containing lipoproteins in the brain as well as periphery, and are putative ligands for TREM2, thus raising the possibility of an APOE-TREM2 interaction modulating different aspects of AD pathology, potentially in an isoform-specific manner. This review is focusing on the interplay between APOE isoforms and TREM2 in association with AD pathology.

## 1. Introduction

Alzheimer’s disease (AD) is the leading cause of dementia worldwide and accounts for 60–80% of all cases [1]. AD is characterized by senile plaques made of β-amyloid peptide (Aβ) and neurofibrillary tangles of hyperphosphorylated tau protein. There are two types of AD: Early-onset familial AD, and late-onset AD (LOAD); LOAD accounting for approximately 95% of all AD cases [2,3]. Familial AD accounts for a small percentage of all cases and occurs exclusively through gene mutations in amyloid precursor protein (APP), or presenilins (PSEN1, PSEN2) that increase the production of Aβ [2,3], or the ratio between longer (Aβ42) and shorter Aβ peptides. These mutations follow a pattern of Mendelian inheritance and result in symptom manifestation before the age of 65 [4]. In contrast, LOAD has no known causative gene mutations, however, genome-wide association studies (GWAS), and whole exome sequencing have identified over 30 AD risk loci [5]. Over half of those have been implicated in innate immune response including Apolipoprotein E (APOE) and triggering receptor expressed on myeloid cells 2 (TREM2) [6,7,8,9].

In humans, the *APOE* gene resides on chromosome 19 and has three alleles with different allele frequencies: *APOEε2*, 5–10%; *APOEε3*, 65–70%; and *APOEε4*, 15–20% [10]. APOE is a 299 amino acid protein, is a major cholesterol carrier in the circulation and the only cholesterol transporter in the brain [11]. In mouse models for AD, the human isoforms APOE2 and APOE3 have the ability to bind and clear Aβ more efficiently compared to APOE4 [12]. The physiological role of APOE in lipid trafficking is crucial as lipids play an essential role in immune regulation through cell signaling, membrane fluidity, and serve as ligands for a number of immune receptors [13]. TREM2 is a cell surface receptor on myeloid cells, and through its interaction with protein tyrosine kinase binding protein (TYROBP), TREM2 activation initiates a multitude of pathways that promote cell survival [14,15], proliferation [16], chemotaxis, and phagocytosis [15,16,17,18,19,20,21], making it vital for normal immune function. The most common TREM2 variant, R47H (arginine to histidine at position 47), impairs ligand binding and increases the risk of developing AD by approximately 4-fold [6,8]. TREM2 has the ability to recognize a variety of ligands, many of them on the surface of apoptotic cells, phospholipids, glycolipids, and lipoproteins including low-density lipoprotein (LDL) and high-density lipoproteins (HDL), Clusterin (APOJ) and APOE [22,23,24]. Emerging evidence suggests that TREM2 can bind to and is a putative receptor for APOE [22,23,24], thus raising the possibility of an APOE-TREM2 interaction modulating AD pathogenesis. This review focuses on the interplay between APOE isoform and TREM2 and their association with AD.

## 2. APOE

### 2.1. APOE Structure and Isoforms

**Figure 1:**
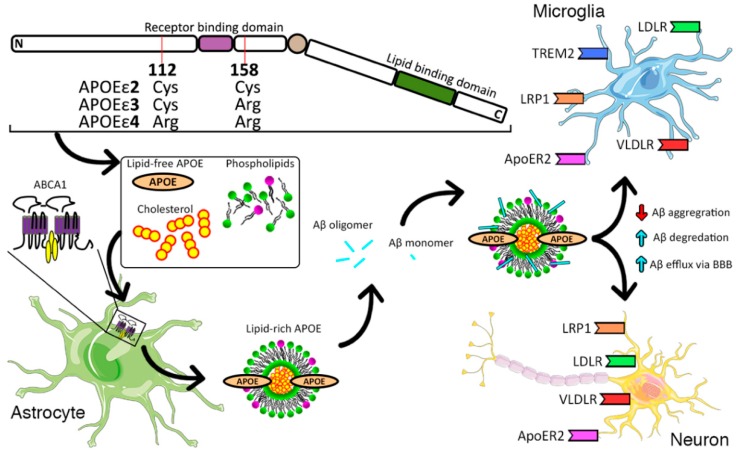
Role of Apolipoprotein E (APOE) in Alzheimer’s disease (AD). In humans, there are three APOE isoforms: APOEε2, APOEε3, and APOEε4. In the brain, APOE is secreted mainly by astrocytes and its lipidation is mediated by ABCA1. ABCA1 transports cholesterol and phospholipids to naïve APOE forming discoidal high-density lipid (HDL) particles. Lipid-rich APOE particles can interact with Aβ monomers and oligomers and bind to the low-density lipid (LDL) receptor family including LRP1, LDLR, VLDLR, and ApoER2 on both neurons and microglia, while also interacting with triggering receptor expressed on myeloid cells 2 (TREM2) only in microglia.

In the brain, APOE is secreted by glia, mainly astrocytes, and is lipidated by adenosine triphosphate-binding cassette transporters A1 (ABCA1) and G1 (ABCG1) (Figure 1). ABCA1 transports cholesterol and phospholipids to lipid-free APOE, thus forming discoidal HDL particles (reviewed in [25,26]). The discoidal HDL particles are composed of 100 to 200 lipid molecules that are surrounded by two apolipoprotein molecules [27]. Once sufficient cholesterol and phospholipids are available to ABCA1, it undergoes a conformation change and forms a dimer. The lipidated dimers interact with actin filaments on the plasma membrane, thereby immobilizing them until lipid-free apolipoprotein directly binds to the ABCA1 dimer. Upon binding, the apolipoprotein accepts the lipids presented by ABCA1 and forms a discoidal HDL particle leaving the ABCA1 dimer to dissociate back to a monomer and begin the process again [27]. In the brain, APOE is primarily synthesized de novo and there is a limited exchange between APOE circulating in the blood and the brain [28,29]. In humans, APOE isoforms differ at either position 112 or 158 (Figure 1). APOE2 has cysteine (Cys) residues at both positions 112 and 158, APOE3 has a Cys residue at 112 and an arginine (Arg) residue at 158, and APOE4 has Arg residues at both positions [30]. All other mammals investigated so far have a single APOE isoform with Arg at the residue equivalent to human APOE 112 [31].

APOE has two functional domains: An N-terminal domain, residues 136–150, and a C-terminal lipid-binding domain, residues 244–272 [10,11]). The N-terminal domain forms a four-helix bundle [32] and the amino acid differences between isoforms alter the protein structure, thus leading to differential lipid and receptor binding. With a Cys residue at position 112, both APOE2 and APOE3 have the ability to form disulfide-linked hetero- and homodimers, while Arg at position 112 of APOE4 significantly impedes the binding [33]. The structural variation between isoforms due to amino acid Cys/Arg at position 158 impacts the receptor-binding domain of APOE and thus, the binding affinity to APOE receptors. The variation at position 112 plays a role in domain–domain interaction and affects lipid binding properties of APOE [34], thus explaining the binding preference of APOE4 for very low-density lipoproteins (VLDL) and APOE3 to HDL [35]. Therefore, the stability and functional role of APOE is largely dependent on its ability to interact with lipids and its receptor binding properties.

### 2.2. APOE Receptors

APOE predominantly binds to receptors of LDL receptor family, which includes low-density lipoprotein receptor (LDLR), LDLR-related receptor 1 (LRP1), very-low-density lipoprotein receptor (VLDLR), and APOE receptor 2 (APOER2) [36,37,38] (Figure 1). The members of the LDL receptor family share structural properties consisting of a short intracellular domain, a transmembrane domain, and a large extracellular domain with a varying number of complement-type repeats, which allow them to interact with APOE [38]. The first identified key member of this family of receptors was LDLR, which is the main receptor for LDL and VLDL. LDLR preferentially binds to lipidated APOE particles, and its deficiency leads to severe hypercholesterolemia and premature atherosclerosis [39]. LRP1 binds to APOE aggregates and is essential for early development, as the deletion of the *Lrp1* gene in mice results in embryonic lethality [40], while the brain-specific knockdown of *Lrp1* inhibits synaptic transmission and motor function [41]. LDLR and LRP1 are the main APOE receptors in the brain, and deletion of *Ldlr* increases APOE levels [42,43]. Both APOER2 and VLDLR are almost exclusively expressed in the brain, are structurally very similar to each other, bind to lipid-free APOE, and are dependent on the extracellular ligand Reelin [44]. In mice deletion of both *Apoer2* and *Vldlr* leads to defective lamination of the cerebellum, cortex, and hippocampus, as well as a reduction in cerebellum volume and impaired motor function [44].

Activation of APOE receptors by Reelin initiates a signaling cascade through the initiation of Src family kinases (SFKs). The activation includes PI3 kinase and Protein kinase B (Akt), which result in reduced phosphorylation of the microtubule stabilizing protein tau, and regulation of microtubule dynamics [45,46]. As noted above, due to the amino acid substitution of Arg with Cys at 158 leading to conformational differences, APOE2 exhibits a severely decreased binding affinity to LDLR (1–2% of APOE3) [47], a significantly decreased affinity to bind LRP1 (40% of APOE3) [47], but similar affinity to VLDLR [48]. The receptors from the LDL receptor family have distinct physiological roles due in part to their affinity to ligands, signaling potency, cellular localization, expression pattern, and endocytosis rate [36].

### 2.3. APOE Function in the CNS

The human brain accounts for approximately 2% of the weight of the body but contains over 20% of its total cholesterol [49]. In the brain, cholesterol is necessary for the formation and maintenance of synapses, and APOE plays a major role in cholesterol homeostasis. The blood–brain barrier (BBB) prevents the exchange between the brain and plasma cholesterol and lipids transported by HDL, LDL, and VLDL [28]. APOE as the major lipid carrier in the brain and has an important role in the transport of cholesterol and other lipids from astrocytes to neurons, where they are needed to maintain synaptic plasticity [50]. The important role of APOE in synaptic integrity and plasticity, as well as dendritic complexity, has been demonstrated by experiments in APOE knockout mice [29,51].

Disruptions in synaptic function such as decreased synaptic density, and alterations in autophagy, are pathological features of neurodegenerative disorders, including AD [52,53,54,55]. There is increasing evidence that APOE isoforms differentially impact synaptic integrity and plasticity [56,57,58,59]. In mice and humans, APOE4 correlates inversely with dendritic spine density [56,60], and synaptic proteins PSD-95, synaptophysin, and syntaxin 1 are altered in an APOE isoform-specific manner (APOE4 < APOE3 < APOE2) [57]. It has been shown that in targeted replacement mice expressing human APOE, APOE4 isoform has a differential effect on neuronal signaling in young and aged mice indicated by the expression level of proteins in NMDAR-dependent ERK/CREB pathway, reduced expression of APOE receptor LRP1 and lower NR2A phosphorylation [59]. Other studies demonstrated that in APOE4 expressing mice, dendritic spine density and complexity, as well as glutamate receptor function, and synaptic plasticity are impaired [61,62]. Meta-analyses addressing the differential effect of APOE isoforms in cognitively healthy adults over the age of 60 suggest that *APOEε4* carriers exhibit impaired episodic memory, executive function, and global cognition, with no impact on primary memory, verbal ability, or attention [63,64]. Studies utilizing the same cognitive tests and similar in size patient cohorts are rare, thus making the findings inconsistent between groups [65]. Whether or not memory and cognitive impairments in humans, carriers of *APOEε4* allele, are associated with a disturbed neuronal signaling and the level of NR2A phosphorylation, as in APOE4 expressing mice, is not known.

## 3. TREM2

### 3.1. TREM2 Structure and Expression

TREM2 is a transmembrane receptor of the immunoglobulin superfamily expressed on the plasma membrane of myeloid cells and microglia, and is active in the innate immune response [66]. TREM2 protein consists of an extracellular Ig-like domain, a transmembrane domain, and a small cytoplasmic tail. In the CNS, TREM2 expression is strongest in the basal ganglia, corpus callosum, spinal cord, and medulla oblongata [67]. Human *TREM2* is located on chromosome 6p21.1 in the *TREM* gene cluster near other TREM and TREM-like genes: *TREML1*, *TREM2*, *TREML2*, *TREML3*, *TREML4*, and *TREM1* [68,69]. Many of these genes are conserved in mice and humans with only *Trem3* and *Trem6* unique to mice and *TREML3* to humans. Both TREM2 and TREM1 interact with TYROBP to initiate pathways involved in cell activation and phagocytosis [16,69]. TREMs proteins are implicated in the clearance of extracellular debris [70].

**Figure 2:**
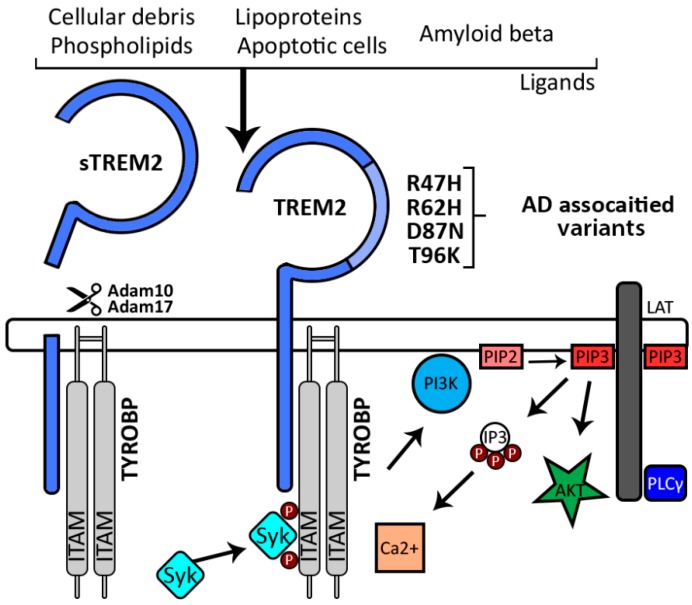
TREM2 activation and downstream signaling. sTREM2 is generated by ADAM10 or ADAM17 mediated proteolytic cleavage. Ligand-activated TREM2 interacts with immune receptor tyrosine-based activation motifs (ITAMs) on TYROBP, which leads to TYROBP phosphorylation and recruitment of spleen tyrosine kinase (SYK). TYROBP/SYK mediated activation of phosphoinositide 3-kinase (PI3K)—AKT pathway and phosphorylation of LAT (linker for activation of T-cells family member 1), recruits other signaling adaptors including phospholipase Cγ (PLCγ). PLCγ degrades phosphatidylinositol-3,4,5-trisphosphate (PIP3) into inositol trisphosphate (IP3), inducing an efflux of Ca^2+^. The ability of TREM2 to bind ligands is influenced by genetic variations, some of which are associated with AD, and located adjacent to or within an electrostatically basic patch (light blue).

The proteolytic cleavage of TREM2 ectodomain generates soluble TREM2 (sTREM2) [71] (Figure 2). sTREM2 has the ability to passes the Brain—cerebral spinal fluid (CSF) barrier and can be detected in CSF [72].

### 3.2. TREM2 Function

TREM2 binds Lipopolysaccharides (LPS) [73], phospholipids [15], HDL [24], LDL, APOE [22,23,24], APOJ [24], apoptotic neurons [18], and Aβ [74] all of which activate signaling pathways (Figure 2). TREM2 conveys intracellular signals through TYROBP, an adaptor protein that contains functional docking sites known as ITAMs. Upon TREM2 activation through ligand binding, the ITAMs on TYROBP are phosphorylated and recruit SYK. SYK activates the PI3K–AKT pathway and phosphorylates the adaptor LAT (linker for activation of T-cells family member 1), which recruits other signaling adaptors including PLCγ. PLCγ degrades PIP3 into IP3, which creates an efflux of Ca^2+^ [15,66,75] (Figure 2).

Unlike the signaling cascade triggered by ligand-activated TREM2 (Figure 2), the biological role of sTREM2 is not well understood. It has been proposed, however, that it either acts as a decoy receptor opposing full-length TREM2 [76] or has another still unidentified function. In cell culture, at least, sTREM2 promoted survival of bone marrow-derived macrophages (BMDM) [77], yet failed to rescue phagocytosis in TREM2-deficient BMDM cells [78].

A well-established function of *TREM2* is the regulation of cell proliferation. Knockdown of *TREM2* in primary microglia leads to a reduction in cell number [79] and TREM2 deficiency inhibits myeloid cell population growth in response to traumatic brain injury [80] and aging [16]. Expression of *TREM2*, even at a normal level, may also impact the proliferation of endothelial cells. Recently, Carbajosa et al. investigated the impact of TREM2 deficiency, in the brain of young and aged mice using RNA-seq and found a disruption of gene networks related to endothelial cells that is more apparent in younger than in older mice. They suggested that the absence of TREM2 in microglia influences endothelial gene expression, which may link immune response and brain vascular disease as an underlying factor in AD pathogenesis [81]. Microglia survival in the context of TREM2 expression has been also linked to the CSF-1-CSF-1R pathway, which is primarily active in conditions of reactive microgliosis [82] and affects Aβ clearance [83]. Since it has been demonstrated that TREM2 signaling, via TYROBOP, synergizes with CSF-1R signaling to promote survival of macrophages [84], a similar mechanism can be involved in microglial survival as well. A recent study by Wang et al. demonstrating that TREM2-deficient microglia exhibited reduced survival at low CSF-1 concentrations support the role of CSF-1R signaling in microglia survival [15]. In conjunction with decreased survival, TREM2-deficient microglia demonstrate a reduced chemotactic capacity. Migration of microglia towards injected apoptotic neurons as well as towards sites of laser-induced damage was also reduced in *Trem2*^−/−^ mice [20].

### 3.3. TREM2 Variants and Neurodegeneration

Rare biallelic mutations that result in loss of function of TREM2 cause Nasu–Hakola disease [67] (NHD) and in some cases Frontotemporal dementia (FTD) [68,85]. NHD is manifested with bone cysts and early onset of neurodegeneration. Brain pathology is comprised of axonal degeneration, white matter loss, cortical atrophy, increased microglia density, and astrogliosis [86,87,88]. The variants associated with NHD and FTD can be a result of coding mutations in the transmembrane domain (D134G, K186N) [67], ectodomain (Y38C, T66M) [68,89,90], early stop codons [91,92], or mutations in a splice site [93,94]. Considering the role of *TREM2* in microglial function, variants in *TREM2* can be part of functional networks involved in multiple neurodegenerative disorders. Numerous studies have evaluated the effect of TREM2 on risk for AD (discussed in Section 4.2), frontotemporal dementia (FTD) [95], amyotrophic lateral sclerosis (ALS) [96,97,98], Lewy body dementia [99], posterior cortical atrophy [100], Creutzfeldt-Jakob disease [101], progressive supranuclear palsy [96], Parkinson′s disease [96], ischemic stroke [96], and multiple system atrophy [102].

TREM2 R47H variant was identified as a risk factor for AD independently by two groups that analyzed European and North American [8], and Icelandic cohorts [6]. Later in the same year, Cruchaga et al. demonstrated that TREM2-R47H variant is associated with a higher level of tau and phospho-tau in CSF [103]. The initial findings for the TREM2-R47H variant were confirmed by other groups [104,105]. In addition, Sims et al. reported a significant association of TREM2-R47H and -R62H variants with LOAD and showed that even after removing these variants from the analysis the association remained significant suggesting the presence of other *TREM2* risk variants [106]. *TREM2 pW191X* and *pL211P* variants were recently identified associated with LOAD in African American cohort but the variants shown to confer AD risk in Caucasians were extremely rare [107]. Similarly, Yu et al. reported several new TREM2 variants in the Han Chinese population, however, none of them was significantly associated with AD risk and the TREM2 R47H variant was not detected in this population [108].

In addition to *TREM2*, another gene in the same cluster—*TREML2* was also examined for association with LOAD. In a meta-analysis study of 36,306 human CSF samples, the missense variant rs3747742 of *TREML2* seemed to confer a protective effect against AD [109]. A complete list of so far identified TREM2 variants—can be found on the ALZ forum website https://www.alzforum.org/.

Recently, Kober et al. demonstrated that NHD variants impact protein stability and decrease TREM2 cell surface expression, while AD variants impact TREM2 ligand binding [110] (Figure 2). When mapping the electrostatic surface of TREM2, Kober et al. identified a large basic patch that was not present in other members of the TREM family [111,112] indicating a unique role for this domain in TREM2 function. Many of the AD-related mutations can be found near or within this basic region of TREM2. Both R47H and R62H decrease the size of the basic patch and reduce binding properties resulting in a loss of function, while T96K increases the size corresponding to a gain of function [110]. 

## 4. APOE, TREM2, and AD

### 4.1. APOE and AD

Studies in mice have suggested that a relationship between APOE isoform and Aβ metabolism was involved in AD pathogenesis. Considering APOE as an Aβ binding protein [113], many of the early in vitro studies tested Aβ binding to APOE and other apolipoproteins [114,115,116,117,118]. While the binding was repeatedly confirmed, none of those studies provided any indication that the risk for AD was dependent on differences in APOE-Aβ binding.

*APOEε4* is the major genetic risk factor for LOAD [119,120]. Inheritance of a single copy of *APOEε4* increased AD risk by ~3-fold, and the inheritance of two copies increases risk by ~12-fold [121]. Compared to AD patients who are not *APOEε4* carriers, AD patients who carry at least one *APOEε4* allele exhibit an earlier disease onset, faster disease progression, and increased brain atrophy [119,122,123]. Importantly, however, homozygous *APOEε3* AD patients still account for the majority of LOAD cases, suggesting that additional genetic or environmental factors are relevant to disease progression. The question, however, if the APOE4 isoform is deleterious or less protective, remains unanswered, with evidence supporting both claims [124]. While the global deletion of APOE is associated with a drastic reduction of compact amyloid plaques in the brain of APP expressing mice [51,125,126,127] the phenotypes of those mice have not been extensively examined to improve our understanding of the role of APOE in the development of AD. Recent studies provided new insight on the role of microglia in the phenotype of APP expressing mice with global deletion of mouse *Apoe*—their reduced microglia recruitment and altered plaque morphology indicated a role beyond APP processing and deposition [128].

Using mouse models for AD, it has been established that human APOE differentially impacts Aβ deposition in a dose-dependent, as well as isoform-specific manner, with APOE4 > APOE3 > APOE2 [12,129,130,131]. Interestingly, recent publications implicated APOE as essential for plaque formation during early seeding stages of Aβ deposition [132,133]. Utilizing APOE3 and APOE4 inducible mice Liu et al. have shown that APOE4 but not APOE3 increases amyloid pathology when expressed during the early seeding stages of amyloid deposition [132]. This impact was not seen in APOE3 mice and was lost when APOE4 was expressed only in later stages of plaque development, indicating APOE4 has the greatest impact on amyloid deposition during the initial seeding stages [132]. By dosing with anti-sense oligonucleotides from birth, Huynh et al. showed a reduction in Aβ deposition in APOE4 mice, whereas there was no effect when the treatment began after the onset of Aβ plaque formation [133]. 

Data from animal models suggest that APOE affects also Aβ clearance in an isoform-dependent manner [12,130], and the lipidation of the protein seems to be of importance [134]. There are two major Aβ clearance pathways in the brain: Receptor-mediated clearance via microglia [135], and astrocytes [136], BBB [137], or through interstitial fluid drainage pathways [138]. Cell facilitated clearance mechanisms are likely to be, in part, mediated by APOE and APOE receptors. APOE receptor-mediated internalization of Aβ seems to be most functional in microglia [139] and astrocytes [140]. ABCA1 functions to alter the lipidation state of APOE in the brain, which consequently impacts Aβ fibrillization (reviewed in [25,26]). In APP transgenic mice, targeted disruption of *Abca1* decreases APOE lipidation and increases amyloid deposition [141,142,143]. Conversely, overexpression of *Abca1* increases APOE lipidation and decreases amyloid deposition [144]. 

The second hallmark of AD, aside from Aβ deposition, is the formation of tau tangles. Early studies demonstrated isoform-specific binding of APOE to human tau in vitro, suggesting an isoform-specific impact on tau pathology [145,146]. Recently, APOE4 has been shown to exacerbate tau-mediated neurodegeneration, while the absence of APOE altogether is protective [147]. Using a P301S tauopathy mouse model on human APOE KI or APOE KO background Shi et al. found no changes at 3 months, but by 9 months the P301S/E4 mice had significantly more brain atrophy than P301S/E2, or P301S/E3, and that APOE KO mice were largely protected from this effect [147].

As a result of the relationship between APOE and AD, it has been suggested that targeting APOE may have a therapeutic potential for AD (reviewed in [148]). There are two potential therapeutic interventions: Regulation of APOE quantity and modification of APOE properties. The former entails the upregulation of APOE levels via liver X receptor (LXR) and PPARγ agonists [149,150,151,152,153]. The administrations of retinoid X receptor (RXR) agonist, bexarotene, was shown to increase APOE level and its lipidation resulting in a reversal of cognitive deficits observed in APP mouse models [60,154,155,156]. However, bexarotene effect on Aβ deposition in AD mouse models is controversial [157,158,159]. Another therapeutic approach is the use of specific antibodies to alter the protein levels of APOE [160]. A recent study demonstrated that using an anti-APOE antibody that recognizes human APOE isoforms, targets, and specifically binds to non-lipidated forms making it effective in reducing amyloid burden in APP transgenic mice [134]. The modulation of APOE properties by structural modification through small molecule correctors [161,162], or by inhibiting APOE-Aβ interactions with small molecule inhibitors [163,164], have also been proposed for therapeutic interventions in AD.

### 4.2. TREM2 and Alzheimer’s Disease

As the resident immune cells of the brain, microglia continuously monitor the brain and respond to damage-related signals that perturb the environment, (reviewed in [165]). The proposed function of microglial recruitment is to form a physical barrier that encapsulates neurotoxic Aβ, thereby restricting plaque growth and containing any neurotoxic effects [166,167]. Deficiency in TREM2 or its adaptor protein TYROBP prevents myeloid cell accumulation around Aβ plaques in a dose-dependent manner [15,166,167,168,169]. In AD patients, heterozygous for the R47H or R62H variants, there are fewer plaque-associated microglia than in those with nonmutant TREM2 [170]. This lack of microglial response in R47H carrying patients has also been shown to increase plaque-associated neuronal dystrophy and reduced microglial coverage [166].

**Figure 3:**
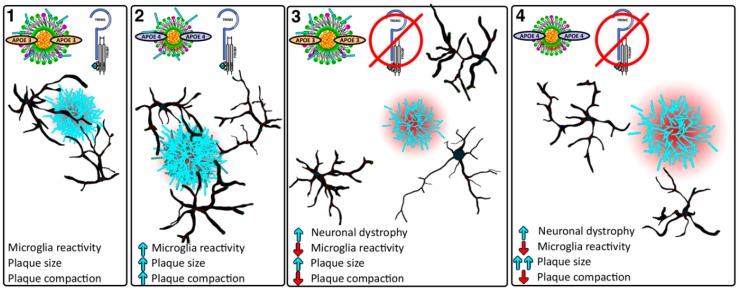
Schematic illustration of the relationship between APOE and TREM2. Microglia in black cluster around amyloid deposits, which impacts plaque morphology and the microenvironment surrounding the plaques. Boxes 1 and 2 illustrate TREM2 in an active state and show an increase in plaque size, compaction, and microglia reactivity in APOE4 compared to APOE3. Microglia which are TREM2 deficient (boxes 3 and 4) fail to contain the plaques allowing them to become more diffuse and increase the surrounding dystrophic area. Arrows are relative to APOE3, TREM2 active (box 1).

Multiple groups have examined the effects of Trem2 deficiency on amyloid pathology with different results based on the mouse model used, as well as the stage of amyloid pathology. Wang et al. examined the effect of TREM2 deficiency in 5XFAD and found that at 8.5 months there was a significant increase of amyloid load in the hippocampus but not in the cortex [15]. Using 5XFAD mice at an earlier age (4 months) the same group found that Aβ accumulation was similar in TREM2 deficient and TREM2-WT 5XFAD mice [167]. Likewise, Jay et al. utilizing APPPS1-21 mice found no change in the amyloid pathology in the cortex and a significant decrease in the hippocampus in *Trem2*^−/−^ mice at 4 months [169]. Interestingly, the same AD mouse model, when examined at 8 months, showed an increased Aβ staining in the cortex and no changes in the hippocampus of *Trem2*^−/−^ mice [171]. Jay et al. concluded that in the early stages of amyloid deposition (2-month cortex, 4-month hippocampus) TREM2 deficiency reduces both plaque number and size, and at later stages (8-month cortex) it increases plaque size and area. Yuan et al. showed that TREM2 deficiency resulted in an increase of diffuse amyloid plaques with longer and more branched amyloid fibrils thus, covering a larger surface area [166]. They conclude that lack of TREM2 may disrupt the microglia barrier around the plaques that regulates amyloid compaction and has a protective role (Figure 3). 

Recently transgenic mouse models expressing TREM2 R47H variant have been generated that demonstrate a diminished response to amyloid deposition exemplified by the reduced cell number and activation of microglia surrounding the plaques [172,173]. These data suggest that TREM2 R47H is a loss of function variant.

In regard to sTREM2, an early study demonstrated that sTREM2 levels were reduced in the CSF of AD patients [19]. However, emerging evidence suggests the opposite: sTREM2 is increased in AD and is positively correlated with tau but not Aβ42 levels [174,175,176,177]. sTREM2 has also been shown to be impacted by TREM2 variants, in which R47H carriers had significantly higher, and T96K, L211P, as well as W199X had significantly lower sTREM2 levels than TREM2 WT controls [176]. A recent meta-analysis study comprising of 17 reports and 1593 patients found sTREM2 levels increased in the early course of AD progression, indicating its potential use as a biomarker for AD progression [72].

### 4.3. APOE, TREM2, and AD

APOEε4 and TREM2-R47H variant were identified as independent risk factors for LOAD [6,119,120,178]. Interestingly both *APOE* and *TREM2* are part of a large group of genes associated with LOAD risk that are expressed in glia cells and related immune response [179]. Several groups have shown that TREM2 binds to APOE using TREM2-Fc fusion pulldown [23], dot blot assays [22], and high throughput protein microarrays [24] (Figure 3). Atagi et al. showed that APOE increases the phagocytosis of apoptotic neurons via the TREM2 pathway and that TREM2 R47H variant was shown to reduce TREM2 affinity to bind APOE [22]. Interestingly APOE lipidation appears to enhance its binding to TREM2 and microglia are more efficient at Aβ uptake when Aβ forms a complex with LDL, APOE, or CLU [24]. In contrast, the same study showed that *TREM2*-deficient microglia have a reduced uptake of Aβ-APOE or Aβ-LDL complexes [24]. A recent study by Jendresen et al. suggests that amino acids 130–149 of human APOE contain the binding site for TREM2, and that there is an APOE-isoform-dependent binding to TREM2 [180]. Although other groups have shown no APOE isoform differences in binding [22,23], possibly due to the sensitivity of binding assays and the lipidation state of APOE.

Microglia as resident macrophages in CNS account for the immune response in the brain, therefore impaired microglia function through either TREM2 deficiency or APOE isoform-specific differences have significant implications. Consistently TREM2 haplodeficient, knockout, or the TREM2 R47H variant, have shown a dose-dependent reduction in microglial activation surrounding amyloid plaques resulting in more diffuse and less compact amyloid plaques. In agreement with these results, overexpression of *TREM2* and increasing TREM2 protein level cause a significant reduction in plaque area, plaque-associated neuronal dystrophy, and amelioration of cognitive deficit in 5xFAD mice [181]. Recent reports identified novel microglia type associated with neurodegenerative diseases (also called disease associated microglia or DAM) characterized by a specific transcriptional profile with both Apoe and Trem2 as part of this program [170,182]. Accordingly, during the progression of neurodegeneration in APP transgenic mice and possible AD brain microglia transcriptome convert from a homeostatic to a disease associated profile. Interestingly, in APP mice that are either TREM2 or APOE deficient microglia fail to convert from a homeostatic into a fully activated state [170,182]. One explanation for these findings may be the significantly decreased plaque load observed in APP transgenic and APOE or TREM2 knockout mice reported by Krasemann et al. [170]. Another explanation is that TREM2 and possibly APOE deficiency prevent microglia conversion from homeostatic to disease-oriented state thus impairing essential defense functions such as chemotaxis, proliferation, phagocytosis, and survival [15,20,128,170,182].

In the end, we can conclude that during the last decade significant progress has been made towards understanding the biology of APOE and TREM2, as well as the biochemical aspects of their interactions and their impact on AD pathogenesis. And although there are still many unanswered questions our knowledge of the most significant risk factors of AD will be soon implemented in successful diagnostic and therapeutic strategies against this devastating disease.

## Abbreviations

AD	Alzheimer’s disease
APOE	Apolipoprotein E
LOAD	Late Onset AD
TREM2	Triggering receptor expressed on myeloid cells 2
Aβ	β-amyloid peptide
EOAD	Early-onset AD
*APP*	Amyloid precursor protein
*PSEN1*	Presenilin 1
*PSEN2*	Presenilin 2
GWAS	Genome-wide association studies
TYROBP	Tyro protein tyrosine kinase binding protein
R47H	Arginine to histidine at position 47
LDL	Low-density lipoprotein
HDL	High-density lipoproteins
APOJ	Clusterin
ABCA1	Adenosine triphosphate-binding cassette transporters A1
ABCG1	Adenosine triphosphate-binding cassette transporters G1
Cys	Cysteine
Arg	Arginine
LDLR	Low-density lipoprotein receptor
LRP1	LDLR-related receptor 1
VLDLR	Very-low-density lipoprotein receptor
APOER2	APOE receptor 2
SFKs	Src family kinases
AKT	Protein kinase B
BBB	Blood–brain barrier
sTREM2	Soluble TREM2
CFS	Cerebral spinal fluid
NHD	Nasu–Hakola disease
FTD	Frontotemporal dementia
ALS	Amyotrophic lateral sclerosis
LPS	Lipopolysaccharide
ITAMs	Immunoreceptor tyrosine-based activation motifs
SYK	Spleen tyrosine kinase
PI3K	Phosphoinositide 3-kinase
PLCγ	Phospholipase Cγ
PIP3	Phosphatidylinositol-3,4,5-trisphosphate
IP3	Inositol trisphosphate
ASO	Anti-sense oligonucleotides
ADSP	Alzheimer’s Disease Sequencing Project
